# Reliability and validity of the Chinese version of Global Sleep Assessment Questionnaire in adult patients with sleep disorders

**DOI:** 10.1371/journal.pone.0356270

**Published:** 2026-08-21

**Authors:** Mingze Sun, Maohuan Peng, Jiahui Gu, Huiqing Liu, Xinru Wang, Chenyang Li, Liyue Xu, Long Zhao, Bing Zhou, Lanbo Wu, Xiaosong Dong, Fang Han

**Affiliations:** 1 Department of Sleep Medicine, Peking University People’s Hospital, Beijing, People’s Republic of China; 2 Capital Medical University School of Nursing, Beijing, People’s Republic of China; Charité - Universitätsmedizin Berlin, GERMANY

## Abstract

**Objective:**

This study aimed to translate Global Sleep Assessment Questionnaire (GSAQ) in Chinese and to validate it in adult patients with sleep disorders.

**Methods:**

GSAQ was translated following the steps of scale translation. Two hundred and one adult patients (aged over 18 years old) with sleep disorders were recruited. All ﬁlled out the 11-item Chinese version of GSAQ. The translated version was tested by exploratory factor analysis (EFA), confirmatory factor analysis (CFA), criterion validity, Cronbach’s α and spilt-half reliability. To evaluate the predictive ability of the questionnaire and elicit cutoff values, receiver operating characteristic (ROC) curves were generated.

**Results:**

Out of the 11 questions in the questionnaire, each item was signiﬁcantly correlated with the total score by a correlation coefﬁcient (r) ranging from 0.24 to 0.73. Four factors were extracted explained 68.89% of the total variance. Cronbach’s α coefﬁcient for the total scale of 11 items was 0.72 and for four factors was 0.81, 0.64, 0.76 and 0.72 respectively, with a split-half reliability of 0.75. ROC analysis yielded an AUC > 0.8. The “obstructive sleep apnea related”, “insomnia related”, “restless legs syndrome related”, and “parasomnias related” scores showed cutoff values of 3,3,1,1, respectively. The GSAQ had positive correlation with AHI (r = 0.248 p = 0.0025), insomnia severity index (r = 0.322, p = 0.017) and the rapid eye movement sleep behavior disorder screening questionnaire (r = 0.721, p < 0.0001). There was no correlation between the total GSAQ score and iRLS (r = 0.140, p = 0.309).

**Conclusion:**

The Chinese version of the GSAQ is available and can be used to evaluate the sleep disorders of adult patients.

## 1. Introduction

Among Chinese individuals aged 15 years and above, the prevalence of sleep disorders is about 53.7% [1]. This, in combination with a consistent association between lower or poor sleep quality and higher burden of multi-morbidity [2], underscores the urgent need to screening for sleep disorders at the primary care level.

At present, there are a variety of single condition measures available for single condition, such as the insomnia severity index (ISI) [3], the STOP-Bang questionnaire [4], the International restless legs syndrome rating scale(IRLS) [5], and the rapid eye movement sleep behavior disorder(RBD) screening questionnaire (RBDSQ) [6]. These instruments, however, were created for use by specialists to assess the severity of specific disorders and are not practical to use in combination for screening.

The well-known, and well-studied, global instrument presently in use is Global Sleep Assessment Questionnaire (GSAQ), which was introduced and validated by Roth et al. in 2002 [7]. GSAQ was also proved its superiority of screening sleep disorders for primary care practice, such as insomnia, OSA, restless legs syndrome(RLS) and parasomnias with a sensitivity of 90.9% and a specificity of 70.58% [8,9]. In parasomnias, rapid eye movement (REM) parasomnias mainly present in adult life [10]. Therefore, our aim in this study is to translate GSAQ into Chinese and validate this version in a sleep clinic.

## 2. Materials and methods

### 2.1. Design

**Fig 1:**
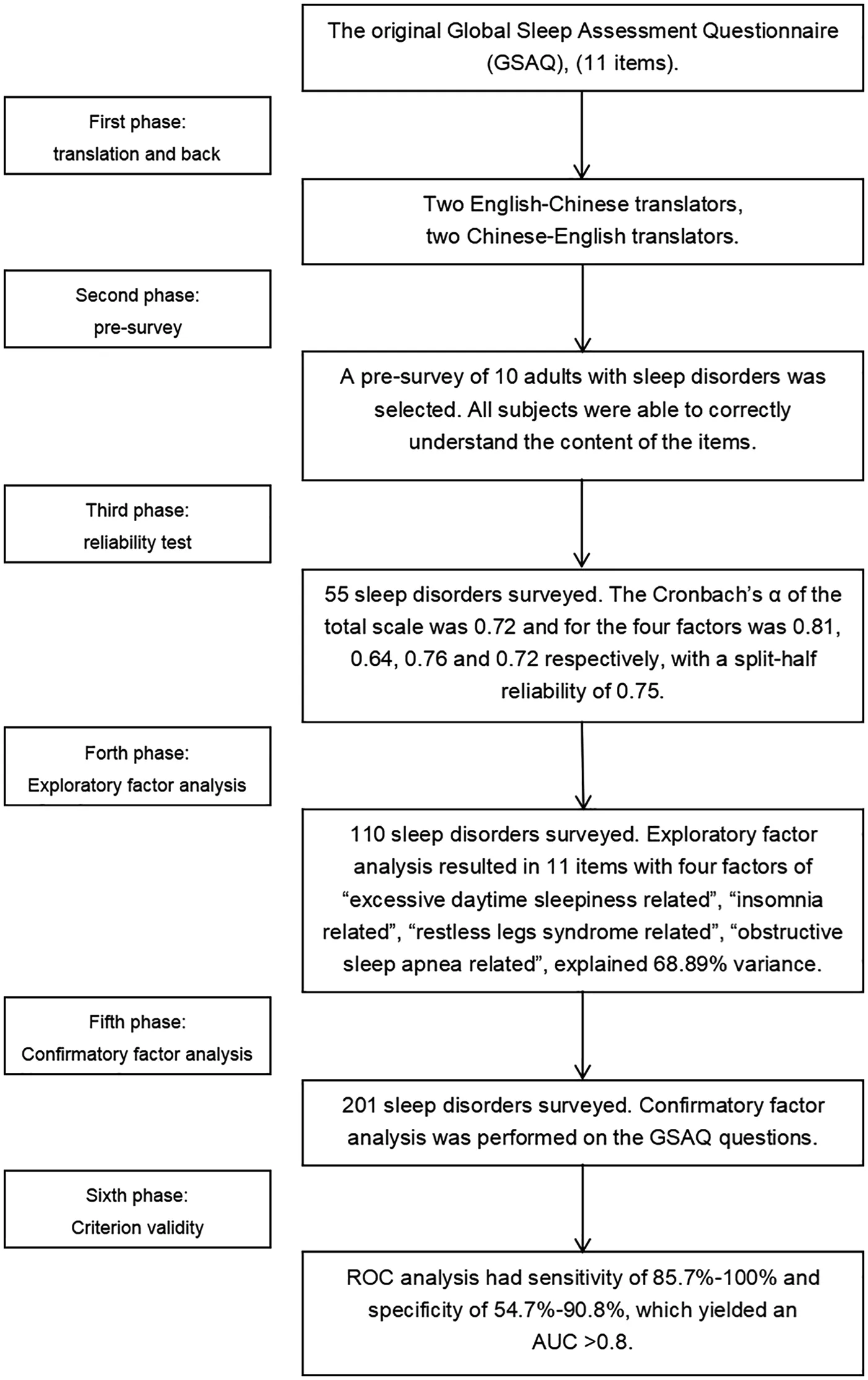
Flow chart of the study.

We performed a cross-cultural adaptation and validation of the GSAQ in a cross-sectional investigation, spanning from November 3^rd^ and December 6^th^, 2024, structured across six phases (Fig 1).

### 2.2. GSAQ translation

GSAQ was translated following the steps of scale translation [11]. Two native Chinese speakers conducted forward translation independently. To estimate the accuracy and fluency of translated GSAQ and discuss any inconsistencies in translations, study panel reviewed and compared the two distinct Chinese versions of GSAQ and finally determined the Chinese version. Two native English translator who had no access to the original English version of GSAQ performed back translation. This new back translation version was compared to the original version. A multidisciplinary expert committee, including methodologists, clinical professionals, all translators, and research team members, subsequently compared the back‑translated versions with the original questionnaire to evaluate semantic, conceptual, content, and cultural equivalence, with all disagreements resolved through group discussion to achieve consensus and finalize a pre‑final Chinese version.

Cognitive debriefing was then carried out with 10 patients, who rated the clarity and cultural appropriateness of each item; items considered unclear by at least 20% of participants were revised, ensuring a minimum inter-rater agreement exceeding 80%. Additionally, an expert panel of ten professionals with expertise in the research topic and target culture evaluated the pre-final version for clarity and content validity, revising items with low relevance. Finally, the revised Chinese questionnaire was administered to the formal study sample to conduct comprehensive psychometric testing, including reliability and validity assessments, to confirm its psychometric properties [11]. As an outcome of this, the final Chinese version of GSAQ was established and approved (S1 Text).

### 2.3. Sample and participants

Since GSAQ consisted of 11 items and the sample size for methodological research was recommended to be 5 times more than the items in the scale, a cross-sectional investigation was performed [12]. We first recruited a convenience sample of 55 with sleep disorders to assess the Chinese GSAQ scale’s internal consistency and split-half reliability. Secondly, an additional 55 patients were collected for exploratory factor analysis (EFA) [11]. Moreover, we obtained more 91 data to perform confirmatory factor analysis (CFA) and calculate cut-off value of different sleep disorders using 201 samples [12]. Finally, to validate the criterion validity of the GSAQ questionnaire, additional 50 patients with parasomnias, 39 patients with RLS and 13 patients with insomnia were reviewed.

All subjects were recruited from the sleep center of Peking University People’s Hospital in Beijing, China. Inclusion criteria were as follows: (a) all of them underwent on nocturnal polysomnography (nPSG), clinical evaluation and were diagnosed with sleep disorders according to ICSD-3 (S2 Text) [13], including OSA, RLS, insomnia and parasomnias, (b) aged over 18 years old, (c) able to read Chinese, and (d) will to participate in this study. Exclusion criteria included combined with other sleep disorders or mental illness or poor mental condition. This study was conducted in accordance with the Declaration of Helsinki. All patients signed the informed consent after obtaining consent, and the privacy rights of human subjects have been observed. The study received approval from the Ethics Committee of Peking University People’s Hospital (Approval No. 2023PHB390−001) and adhered to institutional ethical guidelines.

### 2.4. Data collection

Patients answered the GSAQ, ISI, IRLS, RBDSQ and questions on sociodemographic characteristics, i.e., age, gender, weight, height, onset age, disease duration, education, et al. Moreover, their clinical presentation and results of nPSG were also obtained.

### 2.5. Instruments

The GSAQ is an eleven-item self-reported questionnaire that requires patients to rate on a four-point scale. Extending beyond the present criteria, the response selections were set to frequencies of clinical relevance in order to avoid variance in interpretation between individuals. For example, “never”, “Sometimes”, “Usually”, “Always” could be changed to “0 times per week”, “less than 3 times per week”, “less than 6 times per week”, and “nearly every day”, separately [9]. For ease of interpretation, we described the results of each question ranging from 0(“never”) to 3(“always”). The total score of GSAQ ranged from 0 to 33, the higher the score, the greater likelihood for presence of the sleep disorder. The ISI, IRLS and RBDSQ in Chinese had been proved of good validation and reliability in insomnia [14–16], RLS [17], RBD [18,19], separately.

### 2.6. Data analysis

A descriptive analysis was first performed on the collected data to gain an understanding of the demographic and clinical characteristics of recruited participants. Frequency and percentage were used to express the categorical variables, while mean ± standard deviation (SD) were applied to describe continuous variables.

The final Chinese version of GSAQ were tested for construct validity, including EFA, CFA, and criterion validity. Prior to EFA, the Kaiser-Meyer-Olkin (KMO) and Bartlett’s sphericity test were applied to calculate the adequacy of sampling. In the EFA, principal components factor analyses with Varimax rotation were performed. Factors with a factor loading>0.50 were extracted [20,21]. CFA was also used to further validate the preset model of the scale. The chi-square to degree of freedom ratio (χ2/df), root mean square error of approximation (RMSEA), the goodness-of-fit index (GFI), incremental fit index (IFI) and comparative fit index (CFI) were used to confirm the goodness of fit [22].

Receiver operating characteristic (ROC) curves of the four factors of the questionnaire were generated to find the best cutoff for predicting different sleep disorders. The areas under the curve (AUC) for the four factors score of the questionnaire were calculated. Among several cutoff points, the highest Youden’s J statistic was selected as the optimal cutoff value [23]. Furthermore, Criterion validity was tested with Pearson’s correlation analysis in normal distribution, or Spearman’s correlation coefficient in non-normal distribution. In between the state of “no correlation at all” (*r* = 0) and “perfect correlation” (*r* = 1), interim values of the correlation coefficient were interpreted by convention, values>0.3 might be treated as having correlation [24]. If the respondents answered with a high or low score of more than 15%, indicating that there were floor and ceiling effects [25].

Reliability was assessed by the Cronbach’s α and Spilt-half reliability. The values of 0.7 or higher indicated acceptable reliability [21,22]. Item-analyses to assess the performance of individual item of GSAQ were conducted using critical ratio and correlation analysis [26].

SPSS 29.0 and AMOS 24.0 were used for data analysis. The significance level was set at p-value<0.05.

## 3. Results

### 3.1. Clinical characteristics

**Fig 2:**
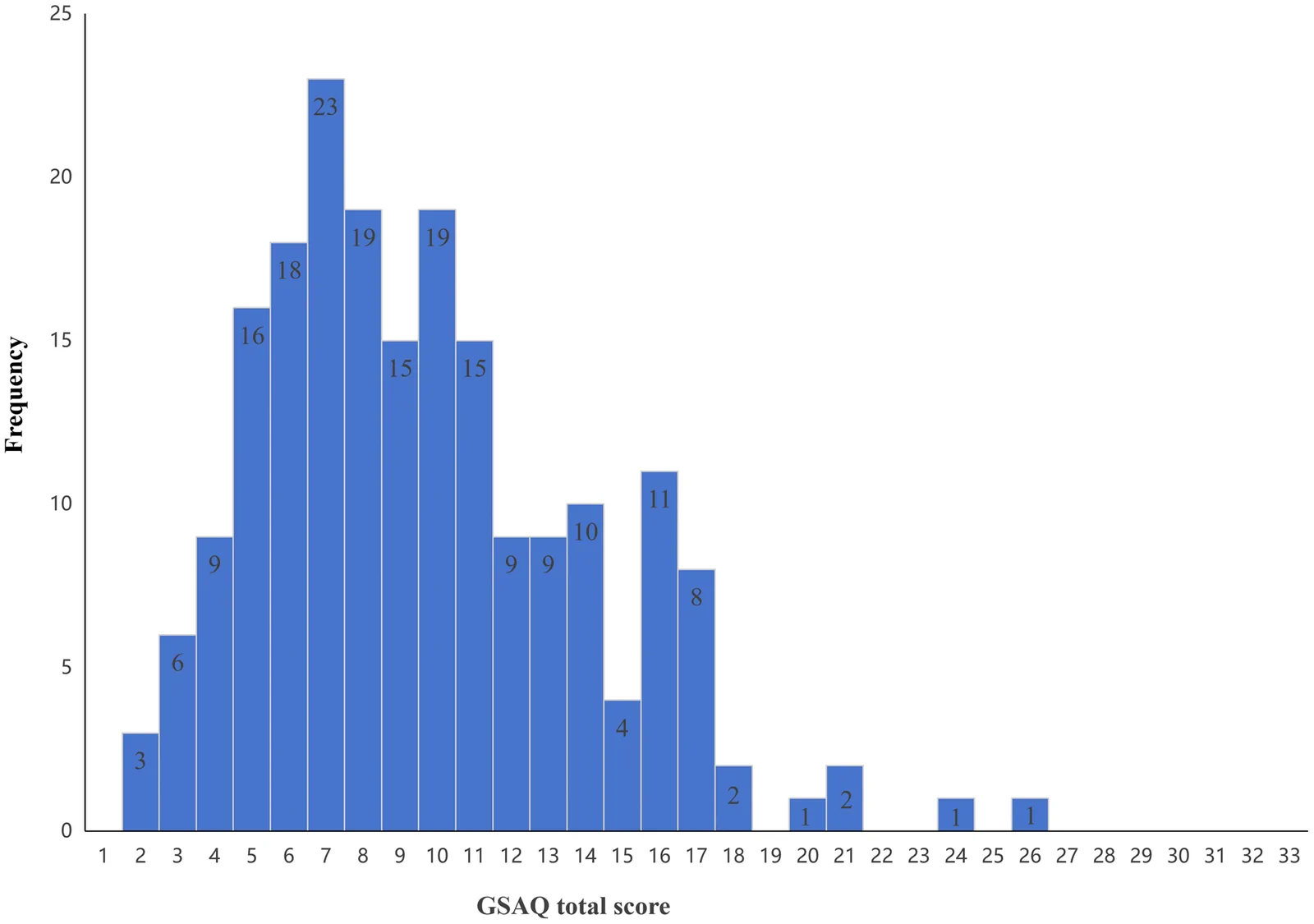
Floor and ceiling results.

A total of 201 patients with sleep disorders completed the Chinese version of GSAQ, with a mean age of 48 ± 15. While many patients presented with only one diagnosed sleep disorder (80.6%), a large percentage (19.4%) of patients presented with and were found to have multiple diagnoses. Of the patients who received multiple diagnoses, 34 patients presented with two, while 5 patients presented with symptoms of more than two. The onset age and disease duration had 36.7 ± 14.8 years and 11.4 ± 10.5 years, respectively. 67.2%(n = 135) were men. Educational levels were below college for 36.8%(n = 74), and college or higher for 63.2%(n = 127). Table 1 presented the characteristics of recruited sample. The scores of Chinese version of GSAQ were well distributed, with a range from 2 to 26. Fig 2 indicated that there were no patients answered with a high or low score of more than 15%, showing the floor and ceiling results were acceptable.

**Table 1:** Sociodemographic and clinical characteristics of the participants.

Characteristic	Overall (N = 201)	OSA (N = 182)	Insomnia (N = 42)	RLS (N = 16)	Parasomnias (N = 5)
Gender					
Male	135(67.2)	129(70.9)	18(42.9)	7(43.8)	2(40.0)
Female	66(32.8)	53(29.1)	24(57.1)	9(56.3)	3(60.0)
Education					
Some college	127(63.2)	115(63.2)	22(52.4)	8(50.0)	2(40.0)
No college	74(36.8)	67(36.8)	20(47.6)	8(50.0)	3(60.0)
Residence status					
Solitary	175(87.1)	161(88.5)	34(81.0)	15(93.7)	4(80.0)
Non-solitary	26(12.9)	21(11.5)	8(19.0)	1(6.3)	1(20.0)
Treatment					
under	27(13.4)	21(11.5)	15(35.7)	6(37.5)	1(20.0)
non	174(86.6)	161(88.5)	27(64.3)	10(62.5)	4(80.0)
Age (years)	48 ± 15	48 ± 15	53 ± 18	56 ± 16	55 ± 18
BMI (kg/m^2^)	27.0 ± 5.0	27.4 ± 5.0	24.4 ± 3.3	25.8 ± 4.6	22.6 ± 3.6
Onset age (years)	36.7 ± 14.8	36.3 ± 14.4	44.3 ± 16.5	48.1 ± 16.4	31.4 ± 21.1
Disease duration (years)	11.4 ± 10.5	11.9 ± 10.6	8.8 ± 9.4	8.1 ± 5.9	24.0 ± 22.5
PSG testing					
Sleep efficiency (%)	79.9 ± 14.5	80.8 ± 13.1	72.9 ± 19.2	69.9 ± 21.8	86.2 ± 7.6
Total sleep time (min)	390.2 ± 83.7	393.9 ± 77.6	361.0 ± 109.3	331.7 ± 113.6	408.6 ± 36.8
AHI (/h)	30.6 ± 26.2	33.6 ± 25.7	11.8 ± 15.0	15.7 ± 27.9	24.3 ± 14.4
Sleep latency (min)	19.9 ± 28.0	19.6 ± 28.1	28.6 ± 43.1	20.3 ± 26.2	6.4 ± 3.3
REM latency	121.3 ± 68.8	120.0 ± 67.8	120.2 ± 72.1	141.3 ± 90.5	184.3 ± 105.0
WASO (min)	78.5 ± 62.0	74.5 ± 56.0	103.2 ± 76.2	116.7 ± 79.4	60.3 ± 37.1
Number of awakening	23.0 ± 14.1	23.1 ± 14.2	22.7 ± 13.7	26.1 ± 13.8	23.2 ± 15.3

**Abbreviations:** OSA, obstructive sleep apnea; RLS, restless legs syndrome; BMI, body mass index; PSG, polysomnography; AHI, apnea hypopnea index; REM, rapid eye movement; WASO, wake after sleep onset.

### 3.2. Item analysis

Each item correlated significantly (p value of all < 0.05) with total GSAQ score, with a correlation coefficient over 0.3 except for item 5 (do you snore loudly). As item 5 is about OSA, and has high clinical significance, we decided to keep this item (Table 2).

**Table 2:** Correlation between total score of GSAQ and each item.

	GSAQ	1	2	3	4	5	6	7	8	9	10	11
GSAQ	1											
1	0.56^b^	1										
2	0.41^b^	0.45^b^	1									
3	0.67^b^	0.23	0.08	1								
4	0.73^b^	0.32^a^	0.18	0.83^b^	1							
5	0.24	−0.26	−0.35^b^	0.19	0.07	1						
6	0.43^b^	−0.08	−0.12	0.10	−0.01	0.56^b^	1					
7	0.47^b^	0.27^a^	0.16	0.04	0.16	0.05	0.19	1				
8	0.43^b^	0.13	0.04	0.13	0.29^a^	0.11	0.13	0.84^b^	1			
9	0.50^b^	0.22	0.19	0.44^b^	0.49^b^	0.07	0.21	−0.02	−0.07	1		
10	0.55^b^	0.21	0.23	0.29^b^	0.36^b^	−0.11	0.14	0.36^b^	0.34^a^	0.21	1	
11	0.53^b^	0.32^a^	0.36^b^	0.22	0.36^b^	−0.02	0.12	0.10	0.04	0.13	0.21	1

**Notes:** ^a^Correlation is significant at the 0.05 level (2-tailed). ^b^Correlation is significant at the 0.01 level (2-tailed).

**Abbreviations:** GSAQ, Global Sleep Assessment Questionnaire.

### 3.3. Validity analysis

#### 3.3.1. EFA and CFA results.

Internal structure validity was verified by applying the EFA using 11 items. The KMO and Bartlett test confirmed that it was fit for applying this technique to this sample (KMO = 0.60, Bartlett’s Test of Sphericity *χ*^2^ = 395.14, p < 0.01). Principal components analysis with Varimax rotation was used to explore the domain of GSAQ. As shown in Table 3. In this step, EFA analysis defined four components of the GSAQ items considering the method of the eigenvalues >1 with 68.89% explained variance.

**Table 3:** Results of the Factor Analysis of Chinese version of GSAQ.

Items	Communalities	Component			
		Factor 1	Factor2	Factor3	Factor4
1	0.47	0.32	**0.59**	0.14	0.08
2	0.73	0.01	**0.83**	0.07	−0.21
3	0.89	**0.94**	0.07	0.06	0.03
4	0.89	**0.92**	0.14	0.13	−0.02
5	0.81	0.08	−0.36	0.01	**0.82**
6	0.87	0.03	0.08	0.07	**0.93**
7	0.82	−0.08	0.11	**0.89**	0.13
8	0.75	0.02	−0.14	**0.85**	−0.07
9	0.40	0.46	0.35	−0.12	0.23
10	0.33	0.26	0.17	0.49	0.03
11	0.62	0.13	**0.77**	−0.03	−0.08
eigenvalues	——	2.79	1.98	1.69	1.13
Cumulative variance percent	——	19.45	37.31	53.80	68.89
KMO	0.60				
Bartlett’s Test of Sphericity	395.14				

**Notes:** Factor 1: excessive daytime sleepiness related; Factor 2: insomnia related; Factor 3: restless legs syndrome (RLS) related; Factor 4: obstructive sleep apnea (OSA) related;

**Abbreviations:** KMO, Kaiser-Meyer-Olkin.

Item 10 exhibited a low communality of 0.33 (<0.4) and factor loading of 0.49 (<0.5), indicating it failed to align with any of the four factors. Nevertheless, Item 10 remained critical for screening symptoms associated with other conditions that impacted sleep quality. Item 9, which addressed abnormal sleep behavior, had a communality of 0.40 and a factor loading of 0.46. Given its relevance to parasomnias and high clinical significance, both Item 9 and Item 10 were retained. In summary, factor 1 was labeled “excessive daytime sleepiness related”, factor 2 was designated “insomnia related”, factor 3 was termed “RLS related”, and factor 4 was designated “OSA related”.

The CFA was performed on the GSAQ questions using AMOS 24.0, showing the goodness of fit of the GSAQ: χ2 = 58.22, P = 0.000, χ2/df = 2,24, RMSEA = 0.08, GFI = 0.94, IFI = 0.93, CFI = 0.92, which all meet the fitting criteria, indicating an acceptable model fit (Table 4) [22].

**Table 4:** Model fit index for the Chinese version of the GSAQ.

Index	χ2/df	RMSEA	GFI	IFI	CFI
Standard value	1-3	0-0.08	>0.9	>0.9	>0.9
Four-factor	2.24	0.08	0.94	0.93	0.92

**Abbreviations:** χ2/df, the chi-square to degree of freedom ratio; RMSEA, root mean square error of approximation; GFI, the goodness-of-fit index; IFI, incremental fit index; CFI, comparative fit index.

#### 3.3.2. Criterion validity.

ROC analysis yielded an AUC > 0.8, indicating that the GSAQ can distinguish excellently between adults with and without sleep disorder. The “OSA related” score and “insomnia related” score showed cutoff values of 3, area under the curve of 0.83, with the specificity of 73.7% and 54.7%, sensitivity of 85.7% and 92.9%, separately. The “RLS related” score and “parasomnia related” score had cutoff value of 1, excellent AUC of 0.90 and 0.92, with the specificity of 90.8% and 71.4%, sensitivity of 87.5% and 100.0%. (Table 5).

**Table 5:** ROC curve for GSAQ scores of four sleep disorders.

Scales	AUC (95%CI)	P value	Cut-off value	Sensitivity (%)	Specificity (%)
OSA	0.83(0.70-0.95)	0.000	3	85.7	73.7
insomnia	0.83(0.76-0.89)	0.000	3	92.9	54.7
RLS	0.90(0.81-1.00)	0.000	1	87.5	90.8
parasomnias	0.92(0.85-1.00)	0.000	1	100.0	71.4

**Abbreviations:** AUC, areas under the curve; CI, confidence interval; OSA, obstructive sleep apnea; RLS, restless legs syndrome.

**Fig 3:**
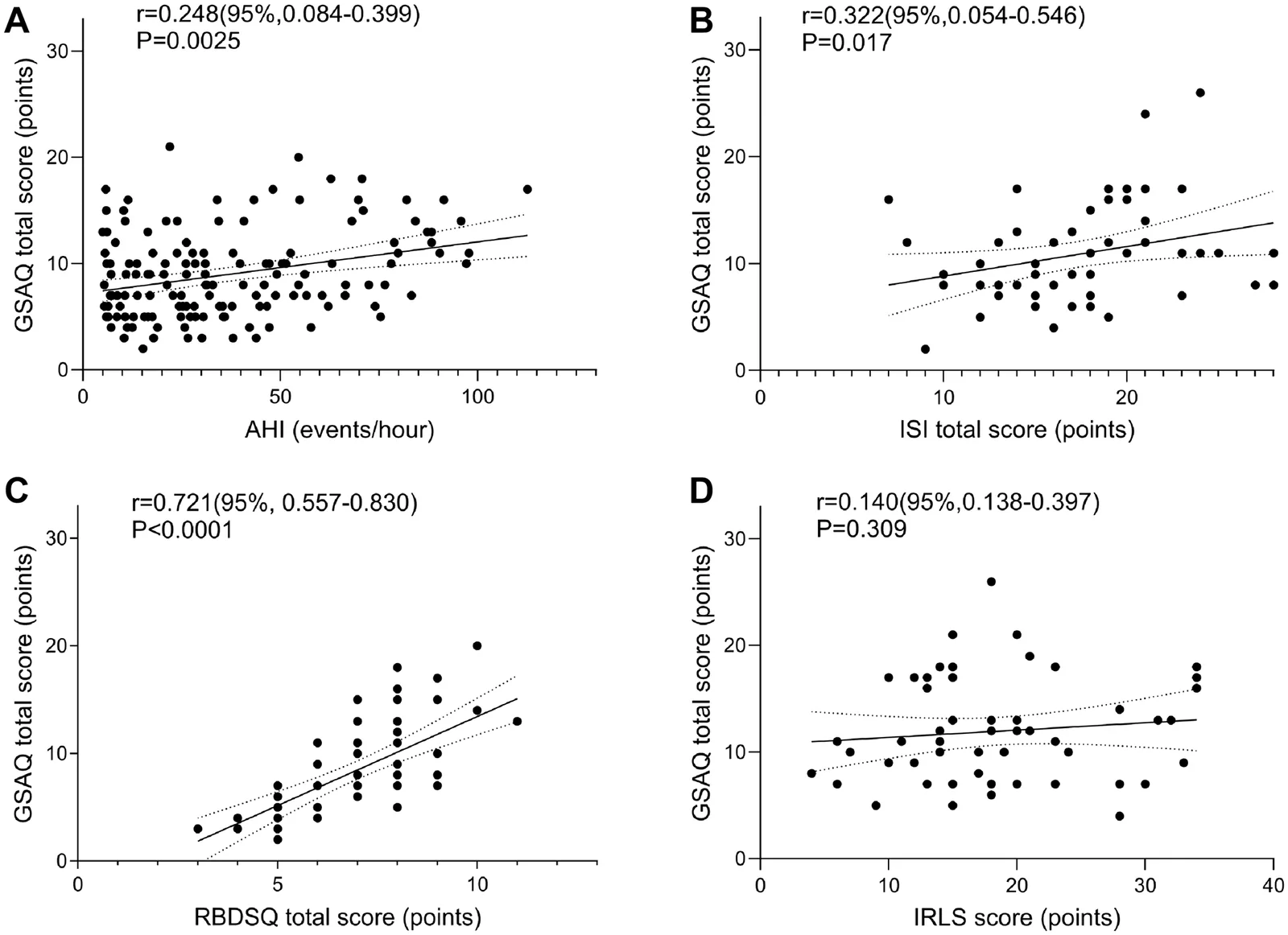
Results of criterion validity. (A) Scatter plot of GSAQ total score and AHI on PSG in the OSA population. (B) Scatter plot of GSAQ total score and ISI total score in the insomnia population. (C) Scatter plot of GSAQ total score and RBDSQ total score in the parasomnias population. (D) Scatter plot of GSAQ total score and IRLS score in the RLS population.

Visual inspection of histograms and Q-Q plots, combined with the Shapiro-Wilk test and Kolmogorov-Smirnov test, confirmed that all datasets were not normally distributed. Spearman’s correlation coefficient were therefore adopted for subsequent analyses. To verify criterion validity in the OSA population, correlations between GSAQ and Apnea Hypopnea Index (AHI) were analyzed (Fig 3). The results showed that the overall GSAQ had positive correlation with AHI (r = 0.248 p = 0.0025). For insomnia and parasomnias, positive correlation was observed between the total GSAQ score with ISI (r = 0.322, p = 0.017) and RBDSQ (r = 0.721, p < 0.0001). However, there was no correlation between the total GSAQ score and iRLS in the RLS population.

### 3.4. Reliability analysis

The Chinese version of the GSAQ exhibited a Cronbach’s α of 0.72 and for the four factors was 0.81, 0.64, 0.76 and 0.72 respectively, with a split-half reliability of 0.75, which indicated high degree of internal consistency and homogeneity.

## 4. Discussion

The present study was carried out to translate and to validate the application of GSAQ in China. All the participants were able to read the our translated Chinese version of GSAQ without any problems, and there is no structural modification to the questionnaire during the adaptation process. The questionnaire is easy to understand and answered within 10 minutes, so that it could be used in the clinics.

The scores of GSAQ, measured by Cronbach’s α coefficient components, showed an adequate internal consistency for each of the 11 items of the questionnaire that screening a particular aspect of sleep disorder. Mahnaz Amini et al. obtained the Cronbach’s α equal to 0.73 in Persion version of GSAQ, which was similar to the present study [7,27].

To the best of our knowledge, this study was the first to exam the factor structure of GSAQ. This is important to determine which factors interfere with the latent variable, so as not to generate an excess of factors that could complicate the analysis unnecessarily. Results was confirmation of four-factor model through principal components factor analyses with Varimax rotation: Factor 1- EDS related (items 3, and 4), Factor 2- insomnia related (items 1, 2, and 11), Factor 3- RLS related (items 7, and 8), and Factor 4- OSA related (items 5, and 6). Our model had salient factor loadings, defined as loadings ≥0.50, for all items except item 9 and item 10. However, both of the two items, mainly screening for parasomnias and other conditions that impacted sleep quality, are important during clinical practice, so they were reserved. Of these, EDS could be a symptom of other sleep disorders [28], 11 questions were obtained for screening four sleep disorders, including OSA, RLS, parasomnia, insomnia. The current study results also support previous studies. A systematic review performed by Klingman et al. [18] in 2017 confirmed sensitivity and specificity values for insomnia, obstructive sleep apnea, restless legs syndrome/periodic limb movement, and parasomnias were based on clinical assessments (the judgment of expert sleep clinicians) [9]. The four-factor model established according to EFA was analyzed through carrying out CFA. The model fitted well and explained the majority of the items. In terms of criterion validity, the “OSA related”, “insomnia related”, “RLS related”, and “parasomnia related” score showed cutoff values of 3,3,1,1 with the sensitivity of 85.7%, 92.9%, 87.5% and 100.0%, separately, which indicates that the GSAQ is an effective and convenient tool for screening the above four different sleep disorders. This is auspicious since the simplicity and accessibility of the GSAQ enable physicians in primary care to identify patients at high risk for specific sleep disorders early and refer them to higher-level hospitals for further evaluation.

Higher GSAQ total scores were associated with a greater probability of having a sleep disorder and were positively correlated with disease severity in patients with OSA, insomnia, and parasomnia. However, in patients with RLS, the total GSAQ score was not proportional to the severity of RLS, probably because GSAQ was initially designed for patients with periodic limb movements instead of RLS [7], and RLS can lead to sleep-onset or sleep-maintenance insomnia [29], then increased scores for factors “insomnia related”. Additionally, RLS maybe overlap with the other sleep disorders such as OSA or insomnia, further influencing the total GSAQ score.

There were some limitations in this study. Firstly, the recruited samples were patients with sleep complaints from a single sleep medicine clinic, which could result in some bias in higher risk and prevalence of sleep disorders. Secondly, all cutoff values were derived solely from internal data without external sample verification. Thirdly, the proportion of patients with parasomnia was relatively small due to the low prevalence rate in general populations. Fourthly, test-retest reliability could not be assessed because the patients were all treated within 1 week. Further studies should conduct in larger sample from primary care and pre-post treatment results could be obtained, thus to confirm the validity of GSAQ in Chinese version.

## 5. Conclusion

GSAQ is a useful tool for screening sleep disorders, designed to identify adults with specific sleep disorders by evaluating the critical values of four key factors. Thus our study provided the availability of the Chinese version of the GSAQ.

## Supporting information

S1 Text Global sleep assessment questionnaire. (DOCX)

S2 Text Diagnostic criteria of ICSD3. (DOCX)

S1 Data Anonymized dataset. (XLSX)
