# Supplementary material for: Reliability and validity of the Chinese version of Global Sleep Assessment Questionnaire in adult patients with sleep disorders
Source: PLoS One. 2026 Aug 21;21(8):e0356270. doi: 10.1371/journal.pone.0356270 (PMC13496455; doi:10.1371/journal.pone.0356270)
Supplement: Supplementary file 1 [file pone.0356270.s001.docx]

**Global sleep assessment questionnaire**

1. Do you have difficulty falling asleep, staying asleep, or do you feel poorly rested in the morning?

- Never
- Sometimes
- Usually
- always

2. Do you fall asleep unintentionally or fight to stay awake during the day?

- Never
- Sometimes
- Usually
- always

3. Do sleep difficulties or daytime sleepiness interfere with your daily activities?

- Never
- Sometimes
- Usually
- always

4. Do work or other activities prevent you from getting enough sleep?

- Never
- Sometimes
- Usually
- always

5. Do you snore loudly?

- Never
- Sometimes
- Usually
- always

6. Do you hold your breath, have breathing pauses, or stop breathing in your sleep?

- Never
- Sometimes
- Usually
- always

7. Do you have restless or “crawling” feelings in your legs at night that go away if you move your legs?

- Never
- Sometimes
- Usually
- always

8. Do you have repeated rhythmic leg jerks or leg twitches during your sleep?

- Never
- Sometimes
- Usually
- always

9. Do you have nightmares or do you scream, walk, punch or kick in your sleep?

- Never
- Sometimes
- Usually
- always

10. Do the following things disturb you in your sleep: Nocturnal cough, nocturnal dyspnea, chest pain/tightness, or wheezing?

- Never
- Sometimes
- Usually
- always

11. Do you feel sad or anxious?

- Never
- Sometimes
- Usually
- always
