# Supplementary material for: Reliability and validity of the Chinese version of Global Sleep Assessment Questionnaire in adult patients with sleep disorders
Source: PLoS One. 2026 Aug 21;21(8):e0356270. doi: 10.1371/journal.pone.0356270 (PMC13496455; doi:10.1371/journal.pone.0356270)
Supplement: Supplementary file 2 [file pone.0356270.s002.docx]

### Diagnostic criteria:

### 1. Obstructive sleep apnea(OSA) (Adult): include either signs/symptoms (eg, associated sleepiness, fatigue, insomnia, snoring, subjective nocturnal respiratory disturbance, or observed apnea) or associated medical or psychiatric disorder (ie, hypertension, coronary artery disease, atrial fibrillation, congestive heart failure, stroke, diabetes, cognitive dysfunction, or mood disorder) coupled with five or more predominantly obstructive respiratory events (obstructive and mixed apneas, hypopneas, or respiratory effort-related arousals) per hour of sleep during PSG. Alternatively, a frequency of obstructive respiratory events ≥ 15/h satisfies the criteria, even in the absence of associated symptoms or disorders.

2. Rapid eye movement sleep behavior disorder(RBD): include (1) repeated episodes of behavior or vocalization that are either documented by PSG to arise from RÉM or are presumed to arise from REM based on reports of dream enactment, and (2) evidence of REM sleep without atonia on PSG (as defined in the scoring manual). When REM sleep without atonia is not observed, the diagnosis may be given on a provisional basis when other clinical findings are strongly suggestive.

3. Restless legs syndrome(RLS): criteria set is based on an urge to move the legs, sometimes accompanied by an uncomfortable sensation that (1) occurs primarily with rest/inactivity; (2) is partially or totally relieved by movement, for as long as the movement occurs; and (3) occurs primarily in the evening or night. Given the somewhat indescribable nature of the symptoms, criteria set emphasizes the importance of ruling out other disorders, the symptoms of which may mimic those of RLS (eg, arthritis, leg cramps, and myalgias).

4. Insomnia: include (1) a report of sleep initiation or maintenance problems, (2) adequate opportunity and circumstances to sleep, and (3) daytime consequences. The ICSD-3 duration criterion for chronic insomnia disorder is 3 months, and a frequency criterion (at least three times per week) has been added.
