# Supplementary material for: Reliability and validity of the Chinese version of Global Sleep Assessment Questionnaire in adult patients with sleep disorders
Source: PLoS One. 2026 Aug 21;21(8):e0356270. doi: 10.1371/journal.pone.0356270 (PMC13496455; doi:10.1371/journal.pone.0356270)
Supplement: Peer review history

# Peer review: Decision Letter 0

**DOI:** 10.1371/journal.pone.0356270.r001

**Authors:** Thomas Penzel

12 May 2026

Dear Dr. Dong,

- **Below, you can see which specific comments the reviewers addressed. As you can see, not all questions require a revision, but some do. Please follow the recommendations.**

plosone@plos.org. When you're ready to submit your revision, log on to https://www.editorialmanager.com/pone/ and select the 'Submissions Needing Revision' folder to locate your manuscript file.

- A letter that responds to each point raised by the academic editor and reviewer(s). You should upload this letter as a separate file labeled 'Response to Reviewers'.
- A marked-up copy of your manuscript that highlights changes made to the original version. You should upload this as a separate file labeled 'Revised Manuscript with Track Changes'.
- An unmarked version of your revised paper without tracked changes. You should upload this as a separate file labeled 'Manuscript'.

If you would like to make changes to your financial disclosure, please include your updated statement in your cover letter. Guidelines for resubmitting your figure files are available below the reviewer comments at the end of this letter.

If applicable, we recommend that you deposit your laboratory protocols in protocols.io to enhance the reproducibility of your results. Protocols.io assigns your protocol its own identifier (DOI) so that it can be cited independently in the future. For instructions see: https://journals.plos.org/plosone/s/submission-guidelines#loc-laboratory-protocols. Additionally, PLOS ONE offers an option for publishing peer-reviewed Lab Protocol articles, which describe protocols hosted on protocols.io. Read more information on sharing protocols at https://plos.org/protocols?utm_medium=editorial-email&utm_source=authorletters&utm_campaign=protocols.

As the corresponding author, your ORCID iD is verified in the submission system and will appear in the published article. PLOS supports the use of ORCID, and we encourage all coauthors to register for an ORCID iD and use it as well. Please encourage your coauthors to verify their ORCID iD within the submission system before final acceptance, as unverified ORCID iDs will not appear in the published article. *Only*  the individual author can complete the verification step; PLOS staff *cannot*  verify ORCID iDs on behalf of authors.

We look forward to receiving your revised manuscript.

Kind regards,

Thomas Penzel

Academic Editor

PLOS One

**Journal Requirements:**

1. When submitting your revision, we need you to address these additional requirements.

Please ensure that your manuscript meets PLOS ONE's style requirements, including those for file naming. The PLOS ONE style templates can be found at

https://journals.plos.org/plosone/s/file?id=wjVg/PLOSOne_formatting_sample_main_body.pdf and

https://journals.plos.org/plosone/s/file?id=ba62/PLOSOne_formatting_sample_title_authors_affiliations.pdf

2. Thank you for stating in your Funding Statement:

This study was supported by the National Key Research and Development Program of China [Project No. 2023YFC3603503]; and National Natural Science Foundation of China[Project No. 82470092].

Please provide an amended statement that declares *all* the funding or sources of support (whether external or internal to your organization) received during this study, as detailed online in our guide for authors at http://journals.plos.org/plosone/s/submit-now.  Please also include the statement “There was no additional external funding received for this study.” in your updated Funding Statement.

Please include your amended Funding Statement within your cover letter. We will change the online submission form on your behalf.

3. Thank you for stating the following financial disclosure:

This study was supported by the National Key Research and Development Program of China [Project No. 2023YFC3603503]; and National Natural Science Foundation of China[Project No. 82470092].

Please state what role the funders took in the study.  If the funders had no role, please state: "The funders had no role in study design, data collection and analysis, decision to publish, or preparation of the manuscript."

If this statement is not correct you must amend it as needed.

Please include this amended Role of Funder statement in your cover letter; we will change the online submission form on your behalf.

4. PLOS requires an ORCID iD for the corresponding author in Editorial Manager on papers submitted after December 6th, 2016. Please ensure that you have an ORCID iD and that it is validated in Editorial Manager. To do this, go to ‘Update my Information’ (in the upper left-hand corner of the main menu), and click on the Fetch/Validate link next to the ORCID field. This will take you to the ORCID site and allow you to create a new iD or authenticate a pre-existing iD in Editorial Manager.

5. Thank you for providing your underlying data as Supporting Information.

We note that the data set contains text or data that is not in English. Please note that PLOS is an English-language publisher, so we require data sets to be provided in English as well. Please upload an English-language version of your data set.

This will also allow us to determine if your data follows PLOS standards per our Data Availability policy here: https://journals.plos.org/plosone/s/data-availability

6. If the reviewer comments include a recommendation to cite specific previously published works, please review and evaluate these publications to determine whether they are relevant and should be cited. There is no requirement to cite these works unless the editor has indicated otherwise. 

**Additional Editor Comments:**

Dear authors,

we have sent the manuscript to two reviewers which provided some valuable details which you find for preparing a revision. Please follow their comments for your submission of a revised version.

Reviewers' comments:

**Comments to the Author**

1. Is the manuscript technically sound, and do the data support the conclusions?

Reviewer #1: Partly

Reviewer #2: Partly

**********

2. Has the statistical analysis been performed appropriately and rigorously?

Reviewer #1: Yes

Reviewer #2: No

**********

3. Have the authors made all data underlying the findings in their manuscript fully available?

The PLOS Data policy

Reviewer #1: Yes

Reviewer #2: No

**********

4. Is the manuscript presented in an intelligible fashion and written in standard English?

Reviewer #1: Yes

Reviewer #2: Yes

**********

**Reviewer #1:**  Sun et al. evaluated the reliability and validity of the Chinese version of the GSAQ. While the study addresses an important topic, there are several methodological concerns regarding the study design.

Major comments:

1. The authors propose the use of the GSAQ in primary care settings. However, the study was conducted in a highly specialized clinic. This raises concerns about the generalizability of the findings. In addition, the proposed cut-off values for OSA-, insomnia-, RLS-, and parasomnia-related domains appear to be entirely data-driven and lack external validation.

2. The authors introduce a novel point-based system to quantify the overall burden of sleep disorders. However, the validity of this approach is unclear. For instance, it is questionable whether symptom frequency (e.g., “nearly every day”) carries equivalent clinical weight across different domains, such as OSA versus parasomnias.

3. In Figure 2, 19 patients are reported to have a total GSAQ score of 10. Without prior expertise in sleep medicine, it is unclear how clinicians, particularly in primary care, would interpret or apply this information in practice. Furthermore, the GSAQ was originally developed for patients with periodic limb movements, rather than restless legs syndrome. Therefore, the absence of an association between GSAQ scores and IRLS scores is not unexpected.

4. The authors report a positive correlation between GSAQ scores and AHI. It would strengthen the manuscript to include ROC analyses for the OSA domain (cut-off 3) across different severity thresholds (AHI ≥5, ≥15, and ≥30), as well as for the insomnia domain (cut-off 3) against clinically relevant ISI thresholds (e.g., ISI ≥15).

Minor comments:

1. Introduction (line 44), it would be appropriate to cite a recent population-based survey on sleep quality in China (PMID: 42004585) which provides relevant epidemiological context.

2. The manuscript states that ICSD-3 criteria were used for clinical diagnosis. Figure 3 indicates that a small number of patients diagnosed with OSA had AHI <5.

**Reviewer #2:** 1. Methods

Translation and cultural adaptationThe authors described forward–back translation but did not provide detailed information on cultural adaptation, cognitive debriefing, expert panel composition, or consensus procedures. Please supplement the full adaptation 流程 following international guidelines (Sousa & Rojjanasrirat, 2011).

Sample size justificationThe justifications for EFA (n = 110) and CFA (n = 201) are unclear. The statement “5 times the number of items” is oversimplified. Please provide explicit sample size rationale with references.

Validity groupingCase–control definitions and gold-standard diagnostic criteria (ICSD-3) for ROC analysis are not fully specified. Please clarify how patients with and without each sleep disorder were defined.

Statistical methods

Normality tests were not reported to justify Pearson or Spearman correlation.

Cutoff values for CFA fit indices (χ²/df, RMSEA, GFI, IFI, CFI) are not provided with references.

Test–retest reliability was not assessed; please clearly state the reason in the limitations.

2. Results

Factor structure and item retentionFour factors were extracted, but factor labeling and item loading assignments are inconsistently presented. Items 9 and 10 showed low communality and factor loadings but were retained. Please provide a clearer rationale in the Results section.

Tables and figures

Table 2 is poorly formatted; please reorganize the correlation matrix for readability.

Figure 3 lacks clear axis labels, units, and confidence intervals.

Complete factor loadings, communalities, eigenvalues, and variance explained should be reported in full.

Scoring and cut-off interpretation

The manuscript reports both total score and four subscale scores. Please clarify whether total score or subscale scores are recommended for clinical screening.

**********

what does this mean?). If published, this will include your full peer review and any attached files.

If you choose “no”, your identity will remain anonymous but your review may still be made public.

**Do you want your identity to be public for this peer review?**  For information about this choice, including consent withdrawal, please see our Privacy Policy

Reviewer #1: No

Reviewer #2: No

**********

[NOTE: If reviewer comments were submitted as an attachment file, they will be attached to this email and accessible via the submission site. Please log into your account, locate the manuscript record, and check for the action link "View Attachments". If this link does not appear, there are no attachment files.]

To ensure your figures meet our technical requirements, please review our figure guidelines: https://journals.plos.org/plosone/s/figures

You may also use PLOS’s free figure tool, NAAS, to help you prepare publication quality figures: https://journals.plos.org/plosone/s/figures#loc-tools-for-figure-preparation.

NAAS will assess whether your figures meet our technical requirements by comparing each figure against our figure specifications.

# Author comment: Author response to Decision Letter 1

**DOI:** 10.1371/journal.pone.0356270.r002

26 Jun 2026

Response Letter to Reviewers’ Comments

Manuscript Number: PONE-D-26-18319

Title: Reliability and validity of the Chinese version of Global Sleep Assessment Questionnaire in adult patients with sleep disorders.

Dear Editor and Reviewers,

We sincerely thank the editor and all reviewers for their valuable comments and suggestions. We have carefully revised the manuscript to enhance its clarity and facilitate the understanding of the readers. Our point-to-point responses are presented in the following, with the editor’s and reviewers’ comments presented in black font, our responses in blue font, and all revisions in the manuscript are highlighted in red. All supporting data for this manuscript are also included in the attachments. We hope that the revision would satisfactorily address the comments and concerns of the editors and reviewers.

Reviewer #1:

Comment 1: The authors propose the use of the GSAQ in primary care settings. However, the study was conducted in a highly specialized clinic. This raises concerns about the generalizability of the findings. In addition, the proposed cut-off values for OSA-, insomnia-, RLS-, and parasomnia-related domains appear to be entirely data-driven and lack external validation.

Response: Thank you for your constructive comments. We respond to the raised concerns point by point as follows:

1. Concern regarding the generalizability of findings: This study aims to evaluate the performance of the GSAQ in Chinese version, with the prospect of promoting its application in sleep clinic and primary care settings. The current investigation was conducted in a specialized sleep center. The enrolled participants presented typical clinical symptoms, which facilitated valid assessment of the scale efficiency and preliminary calculation of cutoff values. We fully acknowledge that the single-center and specialized setting limits the generalizability of our results. The enrolled population cannot fully represent patients seen in primary care facilities, which restricts the direct promotion of our findings to grassroots medical scenarios. This limitation has already been explicitly discussed in the discussion section. Further multi-center studies involving primary care populations will be carried out to verify the practical value of this scale in primary medical environments.�see the fifth paragraph of section “4 Discussion”�

2. Concern about lack of external validation of cutoff values: We agree that the optimal cutoff values for obstructive sleep apnea (OSA), insomnia, restless legs syndrome (RLS) and other sleep disorders were solely determined based on internal data of this study, without external sample verification. Cutoff thresholds derived from a single cohort may carry potential sampling bias and insufficient stability, and thus cannot serve as universal clinical diagnostic criteria. We have supplemented this limitation in the discussion section.�see the fifth paragraph of section “4 Discussion”�

Comment 2: The authors introduce a novel point-based system to quantify the overall burden of sleep disorders. However, the validity of this approach is unclear. For instance, it is questionable whether symptom frequency (e.g., “nearly every day”) carries equivalent clinical weight across different domains, such as OSA versus parasomnias.

Response: Thank you for your constructive comments. This scoring-based system was developed to quantitatively screening sleep disorders. This study verified the four-factor structure of the GSAQ, which corresponds to four sleep disorders: OSA, insomnia, RLS and parasomnias. Symptom frequency was scored separately for each dimension. The optimal cutoff values were determined as 3 for OSA-related symptoms, 3 for insomnia-related symptoms, 1 for RLS-related symptoms and 1 for parasomnia-related symptoms, which can be used to screen individuals at high risk of sleep disorders. We do agree with you that symptom frequency does not carry equivalent clinical significance across different sleep disorders. However, “frequency” is most common used and easily understood when the sleep related complaints were evaluated. Also, the original GSAQ was designed with the structure of “frequency” (never, seldom,...), On this point of view, we keep frequency as a severity scale untill there is other better 指标.

Comment 3: In Figure 2, 19 patients are reported to have a total GSAQ score of 10. Without prior expertise in sleep medicine, it is unclear how clinicians, particularly in primary care, would interpret or apply this information in practice. Furthermore, the GSAQ was originally developed for patients with periodic limb movements, rather than restless legs syndrome. Therefore, the absence of an association between GSAQ scores and IRLS scores is not unexpected.

Response: Thank you for your constructive comments. Fig 2 indicated that no patients answered with a high or low score of more than 15%, showing the floor and ceiling results were acceptable. If the image is ambiguous, we can remove it. Meanwhile, we thank you for your mention that the GSAQ was initially designed for patients with periodic limb movements instead of restless legs syndrome, which can explain our results better. We have added this note to the discussion section.�see the fourth paragraph of section “4 Discussion”�

Comment 4: The authors report a positive correlation between GSAQ scores and AHI. It would strengthen the manuscript to include ROC analyses for the OSA domain (cut-off 3) across different severity thresholds (AHI ≥5, ≥15, and ≥30), as well as for the insomnia domain (cut-off 3) against clinically relevant ISI thresholds (e.g., ISI ≥15).

Response: Thank you for your constructive comments. We have completed the ROC analyses for the OSA domain (cut-off 3) across AHI ≥5, ≥15, ≥30, as well as for the insomnia domain (cut-off 3) against ISI ≥15 as recommended.

Scales AUC (95%CI) Sensitivity (%) Specificity (%)

OSA AHI≥5events/h 0.83(0.70-0.95) 85.7 73.7

AHI≥15events/h 0.69(0.61-0.77) 92.2 41.7

AHI≥30events/h 0.73(0.67-0,80) 96.4 31.4

insomnia ISI≥15 points 0.78(0.69-0.87) 88.9 48.1

We would like to clarify that this scale consists of four factors, each designed to screen for a specific sleep disorder. The score of each dimension is only used for preliminary screening and has no correlation with disease severity. Nevertheless, the total score of the entire scale is significantly associated with the severity of OSA and insomnia. Given the functional positioning of each subdomain, we decide not to supplement additional tables and explanations in the manuscript.

Comment 5: Introduction (line 44), it would be appropriate to cite a recent population-based survey on sleep quality in China (PMID: 42004585IF: 8.1 Q1 ) which provides relevant epidemiological context.

Response: Thank you for the valuable suggestion. We have added the cited population-based survey (PMID: 42004585) to Line 42.

Comment 6: The manuscript states that ICSD-3 criteria were used for clinical diagnosis. Figure 3 indicates that a small number of patients diagnosed with OSA had AHI <5.

Response: Thank you for your valuable comment. We sincerely acknowledge this problematic presentation. Relevant texts and figure annotations will be revised properly to eliminate inconsistency.

Reviewer #2:

Comment 1: Translation and cultural adaptation. The authors described forward–back translation but did not provide detailed information on cultural adaptation, cognitive debriefing, expert panel composition, or consensus procedures. Please supplement the full adaptation 流程 following international guidelines(Sousa & Rojjanasrirat, 2011).

Response: Thank you for your constructive comments. We fully agree that detailed information on cultural adaptation, cognitive debriefing, expert panel composition, and consensus procedures should be clearly described. Following the international guidelines (Sousa & Rojjanasrirat, 2011), we have now supplemented the complete translation and cultural adaptation process in the revised manuscript, including forward–back translation, expert committee review, cultural equivalence evaluation, cognitive debriefing with inter-rater agreement >80%, expert panel validation, and consensus procedures. We appreciate your careful review, which has helped us improve the rigor and transparency of our methods.

Comment 2: Sample size justification�The justifications for EFA (n = 110) and CFA (n = 201) are unclear. The statement “5 times the number of items” is oversimplified. Please provide explicit sample size rationale with references.

Response: Thank you for your constructive comments. We systematically reviewed past papers in your esteemed journal and representative literature, the widely recognized rule of thumb in psychometric research recommends a sample size of 5–10 participants per scale item [1-2]. This criterion is robust and commonly applied in questionnaire development and validation studies in recent years. Representative relevant literatures are attached for reference [3-6]. Since GSAQ consisted of 11 items, We first recruited a convenience sample of 55 with sleep disorders to assess the Chinese GSAQ scale’s internal consistency and split-half reliability. Secondly, an additional 55 patients were collected for exploratory factor analysis (EFA). It strictly meets the 5–10 times item-number principle. For confirmatory factor analysis (CFA), a larger independent sample is required to ensure model fitness and parameter estimation stability. The CFA sample (n=201) exceeds the conventional threshold and fully satisfies the methodological standard for scale structural verification. In addition to the classic subject-to-item ratio rule, this sample size setting is consistent with the sample selection strategy adopted in numerous.

References:

1. Hair JF, Black WC, Babin BJ, Anderson RE. Multivariate Data Analysi . Pearson; New York: 2010. 7th edn.

2. Kalaycı Ş. SPSS Applied Multivariate Statistics Techniques . Asil Yayın Dağıtım; Ankara: 2006. Factor analysis.

3. Lin Y, Li R, Chen Z, Xie Y, Fang J, Li P, Chu M, Liu Y. Validity and reliability of the Dietary Sodium Restriction Questionnaire in peritoneal dialysis patients. PLoS One. 2025 Apr 4;20(4):e0321177. doi: 10.1371/journal.pone.0321177

4. Liu S, Xu D, Wen S, Liu X, Wu D. Cross-cultural validation of the Chinese version of the nurses' ethical decision-making around end-of-life care scale: a cross-sectional study. BMC Nurs. 2026 Feb 24;25(1):178. doi: 10.1186/s12912-026-04450-0.

5. Li C, Xie L, Shang S, Dong X, Wang X, Zhao L, Zhang C, Han F. Narcolepsy Quality-of-Life Instrument with 21 Questions: A Translation and Validation Study in Chinese Pediatric Narcoleptics. Nat Sci Sleep. 2021 Oct 5;13:1701-1710. doi: 10.2147/NSS.S322796.

6. Köroğlu B, Kıralp FSŞ. Validity and Reliability of a Fear of Failure Scale for Adolescents. Alpha Psychiatry. 2025 Apr 21;26(2):39867.

Comment 3: Validity grouping Case–control definitions and gold-standard diagnostic criteria (ICSD-3) for ROC analysis are not fully specified. Please clarify how patients with and without each sleep disorder were defined.

Response: Thank you for your constructive comments. To make the methodology transparent, we have explicitly defined case subjects (patients diagnosed with sleep disorders per ICSD-3) and control subjects (individuals who can not diagnoed with any sleep disorders), In the revised version, we have added complete ICSD-3 diagnostic criteria in the Appendix to address this issue.

Comment 4: Normality tests were not reported to justify Pearson or Spearman correlation. Cutoff values for CFA fit indices (χ²/df, RMSEA, GFI, IFI, CFI) are not provided with references.

Response: Thank you for your constructive comments. We have now supplemented the relevant content in the revised manuscript as suggested:

1. We have added the results of normality tests to verify the rationality of using Pearson and Spearman correlation analyses.�see the second paragraph of section“3.3.2 Criterion Validity”�

2. We have supplemented the commonly accepted cutoff criteria for CFA fit indices (χ²/df, RMSEA, GFI, IFI, CFI) along with corresponding literature references.�see the Table 4 and Reference 22�

Comment 5: Test–retest reliability was not assessed; please clearly state the reason in the limitations.

Response: Thank you for your constructive comments. Test-retest reliability could not be assessed because the patients were all treated within 1 week. The reasons for not assessing test–retest reliability have already been clearly elaborated in the Limitations section. Further studies should conduct in larger sample from primary care and the test–retest results could be obtained, thus to enhance the reliability of GSAQ in Chinese version.�see the fifth paragraph of section“4 Discussion”�

Comment 6: Factor structure and item retention: Four factors were extracted, but factor labeling and item loading assignments are inconsistently presented. Items 9 and 10 showed low communality and factor loadings but were retained. Please provide a clearer rationale in the Results section.

Response: Thank you for your constructive comments. Item 10 remained critical for screening symptoms associated with other conditions that impacted sleep quality. Item 9, which addressed abnormal sleep behavior, had a communality of 0.40 and a factor loading of 0.46. Given its relevance to parasomnias and high clinical significance, both Item 9 and Item 10 were retained. (see the second paragraph of section“3.3.1 EFA and CFA Results” and the third paragraph of section “4 Discussion”).

Comment 7: Table 2 is poorly formatted; please reorganize the correlation matrix for readability.Figure 3 lacks clear axis labels, units, and confidence intervals.

Complete factor loadings, communalities, eigenvalues, and variance explained should be reported in full.

Response: Thank you for your constructive comments. We have carefully revised the relevant contents accordingly:

1. We have fully reorganized the correlation matrix in Table 2 and optimized its overall format to greatly enhance readability, as you suggested.

2. For Figure 3, we have supplemented complete axis labels, corresponding units as well as confidence intervals to make the figure more standardized and informative.

3. Additionally, the complete factor loadings, communalities, eigenvalues and variance explained have been fully reported and presented in Table 3.

Comment 8: The manuscript reports both total score and four sub-scale scores. Please clarify whether total score or sub-scale scores are recommended for clinical screening.

Response: We appreciate your careful review. As clearly stated in the Conclusions section, the four sub-scale scores are recommended for clinical screening. Besides, our study demonstrates that higher GSAQ total scores were associated with a greater probability of having a sleep disorder. Higher GSAQ total scores were positively correlated with disease severity in patients with OSA, insomnia, and parasomnia. However, in patients with RLS, the total GSAQ score was not proportional to the severity of RLS.�see section“5 conclusion” and the fourth paragraph of section “4 Discussion”�.

Attachment Submitted filename: Response to Reviewers.docx

# Peer review: Decision Letter 1

**DOI:** 10.1371/journal.pone.0356270.r003

**Authors:** Thomas Penzel

2 Aug 2026

Reliability and validity of the Chinese version of Global Sleep Assessment Questionnaire in adult patients with sleep disorders.

PONE-D-26-18319R1

Dear Dr. Dong,

We’re pleased to inform you that your manuscript has been judged scientifically suitable for publication and will be formally accepted for publication once it meets all outstanding technical requirements.

Within one week, you’ll receive an e-mail detailing the required amendments. When these have been addressed, you’ll receive a formal acceptance letter and your manuscript will be scheduled for publication.

An invoice will be generated when your article is formally accepted. Please note, if your institution has a publishing partnership with PLOS and your article meets the relevant criteria, all or part of your publication costs will be covered. Please make sure your user information is up-to-date by logging into Editorial Manager at Editorial Manager® and clicking the ‘Update My Information' link at the top of the page. For questions related to billing, please contact billing support.

If your institution or institutions have a press office, please notify them about your upcoming paper to help maximize its impact. If they’ll be preparing press materials, please inform our press team as soon as possible -- no later than 48 hours after receiving the formal acceptance. Your manuscript will remain under strict press embargo until 2 pm Eastern Time on the date of publication. For more information, please contact onepress@plos.org.

Kind regards,

Thomas Penzel

Academic Editor

PLOS One

Additional Editor Comments (optional):

Thank you for answering all questions and queries.

Reviewers' comments:

Reviewer's Responses to Questions

**Comments to the Author**

Reviewer #1: All comments have been addressed

**********

2. Is the manuscript technically sound, and do the data support the conclusions?

Reviewer #1: Yes

**********

3. Has the statistical analysis been performed appropriately and rigorously?

Reviewer #1: Yes

**********

4. Have the authors made all data underlying the findings in their manuscript fully available?

The PLOS Data policy

Reviewer #1: Yes

**********

5. Is the manuscript presented in an intelligible fashion and written in standard English?

Reviewer #1: Yes

**********

Reviewer #1: (No Response)

**********

what does this mean?). If published, this will include your full peer review and any attached files.

If you choose “no”, your identity will remain anonymous but your review may still be made public.

**Do you want your identity to be public for this peer review?**  For information about this choice, including consent withdrawal, please see our Privacy Policy

Reviewer #1: No

**********

# Editor report: Acceptance letter

**DOI:** 10.1371/journal.pone.0356270.r004

**Authors:** Thomas Penzel

PONE-D-26-18319R1

PLOS One

Dear Dr. Dong,

I'm pleased to inform you that your manuscript has been deemed suitable for publication in PLOS One. Congratulations! Your manuscript is now being handed over to our production team.

At this stage, our production department will prepare your paper for publication. This includes ensuring the following:

* All references, tables, and figures are properly cited

* All relevant supporting information is included in the manuscript submission,

* There are no issues that prevent the paper from being properly typeset

You will receive further instructions from the production team, including instructions on how to review your proof when it is ready. Please keep in mind that we are working through a large volume of accepted articles, so please give us a few days to review your paper and let you know the next and final steps.

Lastly, if your institution or institutions have a press office, please let them know about your upcoming paper now to help maximize its impact. If they'll be preparing press materials, please inform our press team within the next 48 hours. Your manuscript will remain under strict press embargo until 2 pm Eastern Time on the date of publication. For more information, please contact onepress@plos.org.

You will receive an invoice from PLOS for your publication fee after your manuscript has reached the completed accept phase. If you receive an email requesting payment before acceptance or for any other service, this may be a phishing scheme. Learn how to identify phishing emails and protect your accounts at https://explore.plos.org/phishing.

If we can help with anything else, please email us at customercare@plos.org.

Thank you for submitting your work to PLOS One and supporting open access.

Kind regards,

PLOS ONE Editorial Office Staff

on behalf of

Dr. Thomas Penzel

Academic Editor

PLOS One
